# PFN2, a novel marker of unfavorable prognosis, is a potential therapeutic target involved in esophageal squamous cell carcinoma

**DOI:** 10.1186/s12967-016-0884-y

**Published:** 2016-05-17

**Authors:** Xiao-bin Cui, Shu-mao Zhang, Yue-xun Xu, Hong-wei Dang, Chun-xia Liu, Liang-hai Wang, Lan Yang, Jian-ming Hu, Wei-hua Liang, Jin-fang Jiang, Na Li, Yong Li, Yun-zhao Chen, Feng Li

**Affiliations:** Department of Pathology and Key Laboratory for Xinjiang Endemic and Ethnic Diseases, Shihezi University School of Medicine, Shihezi, 832002 China; Department of Pathology, Beijing ChaoYang Hospital, Capital Medical University, Beijing, 100020 China; Department of Gynecology, Zhengzhou First People’s Hospital, Zhengzhou, 450000 China; Department of Oncology, The First Affiliated Hospital, Shihezi University School of Medicine, Shihezi, 832002 China; Department of CT and MRI, The First Affiliated Hospital, Shihezi University School of Medicine, Shihezi, 832002 China

**Keywords:** Profilin 2, ESCC, Precursor lesions, Prognosis, Metastasis

## Abstract

**Background:**

Esophageal squamous cell carcinoma (ESCC) is one of the most aggressively malignant tumors with dismal prognosis. Profilin 2 (PFN2) is an actin-binding protein that regulates the dynamics of actin polymerization and plays a key role in cell motility. Recently, PFN2 have emerged as significant regulators of cancer processes. However, the clinical significance and biological function of PFN2 in ESCC remain unclear.

**Methods:**

PFN2 protein expression was validated by immunohistochemistry (IHC) on tissue microarray from Chinese Han and Kazakh populations with ESCC. The associations among PFN2 expression, clinicopathological features, and prognosis of ESCC were analyzed. The effects on cell proliferation, invasion and migration were examined using MTT and Transwell assays. Markers of epithelial–mesenchymal transition (EMT) were detected by Western blot analysis.

**Results:**

Compared with normal esophageal epithelium (NEE), PFN2 protein expression was markedly increased in low-grade intraepithelial neoplasia (LGIN), high-grade intraepithelial neoplasia (HGIN), and ESCC, increased gradually from LGIN to ESCC, and finally reached high grade in HGIN in the Han population. Similarly, PFN2 protein was more overexpressed in ESCC than in NEE in the Kazakh population. The results of Western blot analysis also showed that PFN2 expression was significantly higher in the ESCC tissue than in a matched adjacent non-cancerous tissue. PFN2 expression was positively correlated with invasion depth and lymph node metastasis. High PFN2 expression was significantly correlated with short overall survival (OS) (P = 0.023). Cox regression analysis revealed that PFN2 expression was an independent prognostic factor for poor OS in ESCC. Downregulation of PFN2 inhibited, rather than proliferated, cell invasion and migration, as well as induced an EMT phenotype, including increased expression of epithelial marker E-cadherin, decreased mesenchymal marker Vimentin, Snail, Slug and ZEB1, and morphological changes in ESCC cells in vitro.

**Conclusions:**

Our findings demonstrate that PFN2 has a novel role in promoting ESCC progression and metastasis and portending a poor prognosis, indicating that PFN2 could act as an early biomarker of high-risk population. Targeting PFN2 may offer a promising therapeutic strategy for ESCC treatment.

**Electronic supplementary material:**

The online version of this article (doi:10.1186/s12967-016-0884-y) contains supplementary material, which is available to authorized users.

## Background

Esophageal squamous cell carcinoma (ESCC), a prevalent malignant neoplasm, is the sixth leading cause of cancer-related deaths globally [1, 2]. China has the highest incidence and mortality rate for ESCC, the dominant pathological type of esophageal carcinoma (accounting for 90 %) [3, 4]. Despite the improvement in diagnosis and treatment, the survival of patients with advanced stages of ESCC remains bleak because of early lymph node metastasis, with a 10 % 5 year survival rate for patients [5–7]. In sharp contrast, the 5 year survival rate of patient with early stage of ESCC is higher than 90 % [8]. However, most patients with ESCC are diagnosed at the advanced stage at the time of initial diagnosis. Although various biomarkers have been identified over the past few decades, sensitive and specific molecule markers for early detection and exact indicators for ESCC prognosis are not currently available [9, 10]. Therefore, novel biomarkers that can help in early detection, precise prognosis and individualized therapy of ESCC patients are needed.

Profilins are 12–15 kDa actin-binding proteins found in eukaryotes [11]. These proteins sequester actin monomer protein and regulate the dynamics of actin polymerization [12, 13]. In mammals, four discrete profilin genes (PFN1, PFN2, PFN3, and PFN4) have been identified. PFN1, the mainstay of the profilin family, is ubiquitously expressed in all cell types, except skeletal muscle [13, 14]. In amniotes, two alternatively spliced variants of PFN2 (PFN2a and PFN2b) have been identified. PFN2a is a major isoform that is predominantly expressed in neuronal cells. Isoform PFN2b, of which mRNA is detectable in a finite number of adult tissues mainly in the kidney, is rare [15, 16]. PFN3 and PFN4 expressions are limited to the testis [17, 18]. In general, PFN1 and PFN2, as the most common profilins in mammalian cells, display similar actin monomer-binding properties [19]. Profilin accelerates the extension of the uncapped actin filament in the presence of formin and then depolymerizes the capped actin filament, thereby leading to cytoskeletal changes [20, 21]. In addition, the disruption of profilin by gene deletion of PFN1 in null mice [22] or by anti-profilin transfection in bovine oocytes [23], results in early embryonic death. In response to depolarization, those mice lacking PFN2 exhibited a hindered synaptic actin polymerization [24]. These phenomena indicate the non-redundant roles of PFN1 and PFN2 in vertebrate gastrulation.

In recent years, the properties of the profilin family that is involved tumors in progression have been reported. PFN1 has been viewed as a negative regulator for the migration and invasion of breast cancer cells [25–27]. However, a recent report highlighted the opposite roles of PFN1 and PFN2 in the membrane protrusion, cell migration and invasion of breast cancer cells [28]. Another recent report showed that PFN1 had contrasting effects on breast cancer in a context-dependent manner [29]. Other reports suggested that post-translational modifications, such as phosphorylation of PFN1 at Ser 137 or Tyr 129, could change the properties of cancer cells [30–32]. Hence, the roles of profilin in various cancers need to be further clarified. Similarly, the biological function of PFN2 in digestive tract tumors remains controversial. In oral squamous cell carcinoma (OSCC), PFN2 inhibits tumor growth and aggressiveness [33]. Nevertheless, PFN2 significantly enhanced migration and invasion ability of HT29 human colorectal cancer stem cells [34]. However, only few studies have investigated the role of PFN2 in ESCC progression.

In this study, we explored the expression of PFN2 in ESCC and precursor lesions from two different races and then analyzed the associations of PFN2 expression with the clinicopathologic features of ESCC. Functional studies were performed to identify the undiscovered biological function of PFN2 in ESCC cells. In sum, PFN2 assumes a tumor-promoting role in ESCC progression and metastasis by inducing EMT. PFN2 may also function as an independent prognostic biomarker and chemotherapeutic target for ESCC patients from Han and Kazakh ethnic populations, particularly those who are suffering from early stages of cancer.

## Methods

### Patients and tissue specimens

The ESCC tissues used for IHC staining of PFN2 were gathered from two separate groups of ESCC. One group comprised 192 Han Chinese patients with ESCC collected between 1997 and 2013 from the First University Hospital, Shihezi University School of Medicine. The other group included 168 Kazakh ESCC patients whose data were gathered between 1984 and 2011 from Xinjiang Yili Prefecture Friendship Hospital, People’s Hospital of Xinjiang Uyghur Autonomous Region, and the First University Hospital, Shihezi University School of Medicine. No restrictions regarding sex, age, or stage of disease were placed. Except for diagnostic biopsies, iatrochemistry, or radiotherapy, none of the patients received prior surgery. Data on clinicopathologic parameters, such as invasion depth and lymph node metastasis, were collected. Tumor node metastasis (TNM) stages were assessed for each case in accordance with the seventh edition of the AJCC cancer-staging manual [35]. All patients involved in the study gave their written informed consent, and the research protocol used in this study was approved by the medical ethics and human clinical trial committee of the Shihezi University School of Medicine.

The clinical characteristics of patients from both validation centers are listed in Table 1. Immediately after surgery, 192 cancerous tissues, together with their 135 adjacent mucosa, were collected from the Han patient group. A total of 168 cancerous tissues, with their 55 adjacent mucosa, were collected from the Kazakh patient group. The tissues were fixed in 10 % formalin for 24 h and then embedded in paraffin. Moreover, from the Han group samples, 85 precursor lesions, called esophageal squamous intraepithelial neoplasia (ESIN), were selected, including 52 LGIN and 33 HGIN. The diagnoses of all the subjects were from two pathologists independently. Clinical and pathological information of all cases were acquired from medical records. Follow-ups were conducted on 71 Han patients by phone or other methods, with a follow-up deadline of 10 July 2015.

**Table 1:** PFN2 clinicopathological characteristics of patients with ESCC from Chinese Han and Kazakh population

Characteristic	Han ethnic		Kazakh ethnic	
	(N = 192)		(N = 168)	
	No	%	No	%
Age at surgery, years				
Median	62.5		57.4	
Range	42–81		34–75	
Gender				
Male	137	71.4	108	64.3
Female	55	28.6	60	35.7
Differentiation^a^				
Well	67	34.9	39	23.2
Moderate + Poor	125	65.1	129	76.8
Invasion depth				
T1–T2	79	41.1	83	49.4
T3–T4	113	58.9	85	50.6
Lymph node metastasis				
No	127	66.1	79	47.0
Yes	65	33.9	89	53.0
TNM stage^b^				
I + II	154	80.2	111	66.1
III + IV	38	19.8	57	33.9

^a^Histologic grade was based on WHO classification published in 2010

^b^TNM stage was assessed according to the 7th edition of the AJCC Cancer Staging Manual

### Tissue microarray fabrication and immunohistochemistry analysis

Tissue microarray (TMA) construction and IHC process were implemented according to previous research [36]. In brief, paraffin-embedded samples was first cut into 4-μm-thick slices and stained using hematoxylin and eosin (HE). Many cancerous regions of each sample were distinguished from HE-stained slides. Subsequently, the aforementioned areas were located to the ready paraffin block for TMA fabrication. Then, we bore a hole in these regions and inserted into a recipient paraffin block using a tissue arrayer with a hollow needle with a diameter of 1.0 mm (ALPHELYS, Plaisir, France). Finally, serial slices were built from the TMA blocks for IHC staining.

PFN2 protein expression was detected using the Envision system (Dako, Carpinteria, CA). Next, the tissue slices were prepared on microslides and baked at 65 °C for 2 h and deparaffinized using xylene and rehydrated using alcohol. Then, the slices were immersed in hydrogen peroxide (3 %) for 10 min to eliminate endogenous peroxidase. Subsequently, antigen retrieval was implemented by heating the tissue slices in a pressure cooker under high power for 10 min in a citrate buffer solution. The tissue sections were then incubated with anti-PFN2 antibody (ab55611; Abcam, Cambridge, MA; dilution 1: 400) at 4 °C overnight. By contrast, negative control sections were incubated with PBS. The next day, the slides were incubated with a second antibody for 30 min at 37 °C. Afterward, these slices were covered with a fresh diaminobenzidine solution then stained with hematoxylin and dehydrated with graded alcohol and xylene. Finally, neutral balsam (ZSGB-Bio, Beijing, China) and coverslips were added, and the slices were air dried before the analysis.

### Semi-quantitative assessment and scoring

With respect to the assessment of IHC results, we referred to a previous scoring method [37]. The expression of PFN2 was semi-quantitatively scored on the basis of the percentage of positive cells and cytoplasmic/nuclear staining intensity. The percentage of positive cells were 0 (<5 % positive cells), 1 (6–25 % positive cells), 2 (26–50 % positive cells), 3 (51–75 % positive cells), or 4 (>75 % positive cells). The cytoplasmic/nuclear staining intensity was classified into the following: 0 score (negative), 1 (light yellow), 2 (yellow), and 3 (brown). The percentage of positive cells and staining intensity were then multiplied to obtain the IHC score (IS) for each patient. All immunostained results were assessed by two pathologists independently and finally assigned a consistent score. If the two pathologists disagreed over the immunohistochemical results, a third pathologist was invited to assist in analyzing the results. Thus, the IS ranged from 0 to 12 scores. The optimal cut-off values for the evaluation system can be perceived as follows: high expression of PFN2 was defined as an expression index score of ≥4, and low expression of PFN2 was defined as an expression index score of <4. These cases were divided into two groups based on their IS of PFN2 staining. Cases with scores of ≥4 were categorized as the high expression group, and cases with a score of <4 were categorized as the low expression group.

### Cell culture and cell transfection

Three EC cell lines (EC9706, Eca109, TE-1) were obtained from the Institute of Biochemistry and Cell Biology of the Chinese Academy of Sciences. Cell culture was performed in DMEM Medium (GIBCO-BRL) in addition with 10 % fetal calf serum, 100 mg/mL streptomycin, and 100 U/mL penicillin in moist air containing 5 % CO_2_ at 37 °C. Oligonucleotide small interfering RNA (siRNA) duplexes were synthesized by GenePharma (Shanghai, China). The following siRNA sequence for PFN2 was used: 5′-CAU AUG AAC UCG CUU UAU A-3′. A non-target scrambled siRNA was used as the negative control: 5′-UUC UCC GAA CGU GUC ACG UTT-A-3′. The cells were transfected with 130 nM siRNA targeting PFN2 or RNAi negative control duplexes using HiPerFect transfection reagents (Qiagen, Hilden, Germany) in serum-free conditions according to the Quick-Start Protocol.

### Western blot analysis

Western blot analyses were performed on cell lysate prepared from three esophageal cancer cell lines and esophageal tissue as described previously. Equal amount of protein lysate were loaded and separated on 10 % SDS–polyacrylamide gel electrophoresis and then transferred to a PVDF membrane (Millipore, USA). After being blocked by 5 % fat-free milk in TBS buffer, membranes were probed with the following antibodies overnight at 4 °C: mouse monoclonal anti-PFN2 (Abcam; 1:1000 dilution), rabbit anti-E-cadherin (Santa Cruz; 1:200 dilution), rabbit anti-Vimentin (Abcam; 1:1000 dilution), rabbit anti-Snail (Proteintech; 1:500 dilution), rabbit anti-Slug (Proteintech; 1:200 dilution), rabbit anti-ZEB1 (Proteintech; 1:500 dilution) and mouse anti-β-actin (Zhongshan Biotechnology; 1:1000). Bound antibodies were detected with secondary HRP-conjugated antibodies (Santa Cruz) for 2 h at room temperature. After washing, the resulting bands were visualized using the standard ECL procedure (Kangwei, Beijing). Images were acquired using the image acquisition system (BioRad, USA) and their grayscale value was analyzed by the image analysis program (Gel-Pro Analyzer 4.0, USA).

### Measurement of cell proliferation

Cells (4 × 10^3^ cells/well) were seeded at 96-well plates in triplicate. The cells were incubated for 24, 48, and 72 h, and were then stained with 20 μL 3-(4,5-dimethylthiazol-2-yl)-2,5-diphenyltetrazolium bromide (MTT) dye (5 mg/mL, Solarbio) for 4 h. Subsequently, supernatants were removed, and 150 μL of DMSO was added to each well. The plates were shook rapidly until the formazan crystals dissolved completely. The absorbance value of each well was detected at 490 nm using a spectrophotometer (Bio-Rad, USA). Independent experiments were repeated three times.

### Cell migration and invasion assays

Cell migration and invasion assays were performed as previously described [37]. Migration assay was performed by using Transwell insert chambers (Corning, USA). Transfected cells measuring 8 × 10^4^ were plated onto the top surface of the chamber in triplicate. After incubation for 24 h under suitable conditions, non-invaded cells were removed, and the membranes containing the invaded cells were fixed and stained with 0.1 % crystal violet. More than six fields were counted for each sample. Three independent experiments were performed. For the cell invasion assay, the process was analogous to the cell migration assay, except for that the membranes were smeared with Matrigel (BD Biosciences, USA) and the seeded cells were incubated for 8 h.

### Statistical analysis

Statistical analysis of the experimental data was performed using SPSS 19.0 software (IBM Corporation, Armonk, NY, USA). Categorical data were compared using χ2 test and Fisher’s exact test to analyze the differences in PFN2 expression and clinical characteristics. The sensitivity and specificity of ESCC and precancerosis forecast were assessed by receiver operating characteristic (ROC) curves. Survival curves were analyzed using the Kaplan–Meier method. A Cox regression model was used to analyze the independent prognostic factors. All statistical results were demonstrated using GraphPad Prism 5 software. For all tests, the P values of less than 0.05 were considered statistically significant.

## Results

### PFN2 is overexpressed in ESIN and ESCC tissue compared with NEE tissue

**Fig. 1:**
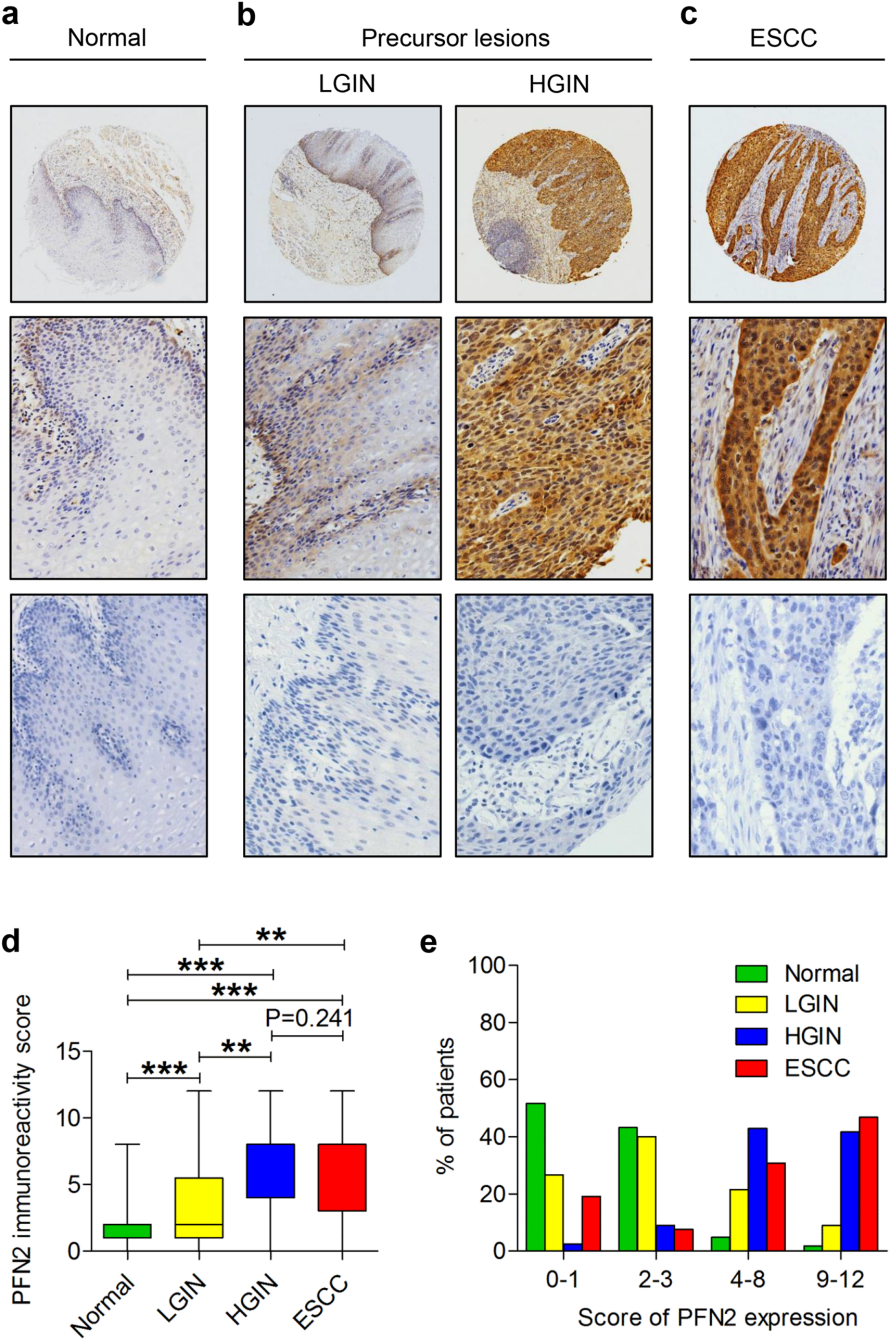
IHC analysis of PFN2 protein in adjacent normal tissues, precancerous lesions, and ESCC tissues from Chinese Han population. Typical PFN2 immunostaining in **a** NEE, **b** LGIN and HGIN, and **c** ESCC in the Chinese Han population (*top image* magnification ×40; *middle* and *bottom* image magnification ×200; *bottom image* stands for negative control) PFN2 staining was located to the nuclei/cytoplasm. **d** (*Box plot*) Range of PFN2 immunoreactivity score in NEE, LGIN, HGIN, and ESCC tissues (**P < 0.01, ***P < 0.001). **e** (*Bar chart*) Distribution ratio of NEE, LGIN, HGIN and ESCC tissues in four-level score (0–1, 2–3, 4–8, and 9–12) of PFN2 expression

**Fig. 2:**
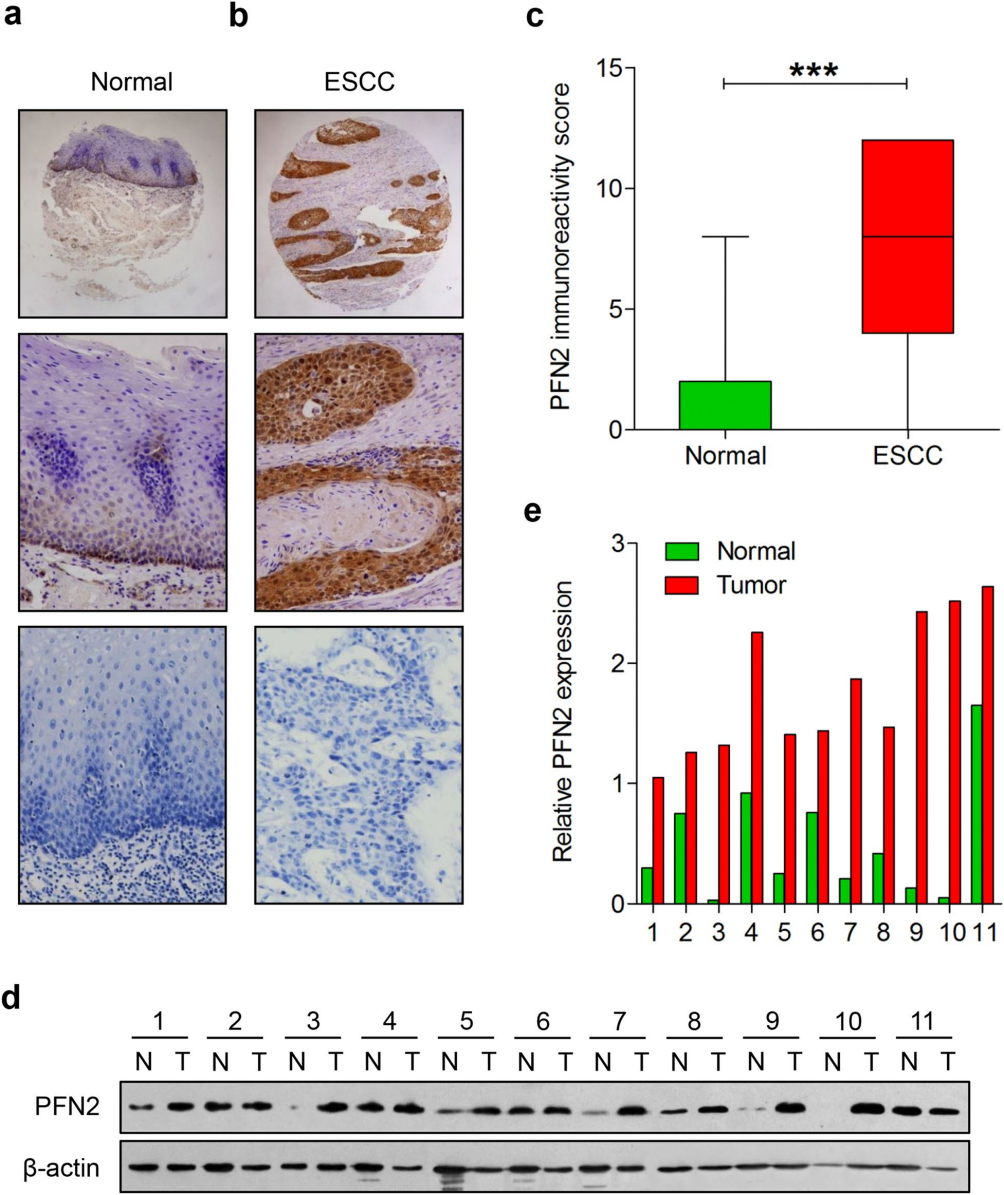
IHC analysis of PFN2 protein in adjacent normal tissues and ESCC tissues from Kazakh population. Typical PFN2 immunostaining in **a** NEE and **b** ESCC in the Chinese Kazakh population (*top image* magnification ×40; *middle and bottom image* magnification ×200; *bottom image* stands for negative control). PFN2 staining was localized to the nuclei/cytoplasm. **c** (*Box plot*) Range of PFN2 expression score in NEE and ESCC tissues (***P < 0.001). **d** PFN2 expression at the protein level was detected by Western blot analysis in 11 matched pairs of ESCC tissues (T) and adjacent normal tissues (N). β-actin was used as an endogenous control. **e** *Bar chart* showing the relative PFN2 protein expression level to β-actin in ESCC tissues and paired NEE tissues (**P < 0.01)

To determine the clinical relevance of PFN2 and explore its potential role in ESCC progression, we detected the expression of PFN2 protein by IHC staining in NEE, LGIN, HGIN, and ESCC tissues gathered from the Chinese Han and Kazakh populations. The IHC results revealed that PFN2 staining was mainly located in the nuclei/cytoplasm, and the distribution of PFN2 staining was significantly different during the different development phases of ESCC. For the Han population, the IHC analysis displayed that the frequency of PFN2 protein overexpression was lowest in the NEE tissue (8.1 %), but its frequency increased gradually along with the progress of esophageal cancer, with 40.4 % (21/52) of LGIN and 91.0 % (30/33) of HGIN. Meanwhile, a slight decrease in frequency was observed at 82.8 % (159/192) of ESCC, but a high expression level of PFN2 protein was still observed. In NEE, PFN2 staining was weak or negative and was predominantly observed in the nuclei/cytoplasm of basal cells (Fig. 1a); however, PFN2 was expressed in HGIN and ESCC cells with the strongest signal in nuclei/cytoplasm (Fig. 1b, c). The tendency of PFN2 overexpression rates exhibited a progressive increase in NEE, LGIN, and ESCC, and peaked in HGIN (Fig. 1d). Moreover, the four-level score (0–1, 2–3, 4–8, and 9–12) distribution of PFN2 protein expression in the different development stage of ESCC was significantly distinct (Fig. 1e). An in-depth analysis uncovered that the overexpression rates of PFN2 protein increased significantly in LGIN, HGIN, and ESCC compared with that in NEE (P < 0.001). Meanwhile, significant differences were observed between LGIN and HGIN (P < 0.001), as well as between LGIN and ESCC (P < 0.001). PFN2 overexpression was highest in HGIN, not ESCC. However, the difference between these two subgroups was not statistically significant (P > 0.05, Table 2). The expression of PFN2 protein was also analyzed in the Kazakh patients. The statistical analysis showed that the staining density of PFN2 in ESCC tissue was distinctly stronger than that in adjacent normal tissue in the Kazakh population (P < 0.001, Fig. 2a, b, c). High PFN2 expression was detected in 146/168 (86.9 %) of ESCC tissues and only in 8/55 (14.5 %) of normal samples. The results were constant the findings from the Han patients, which indicated no differences with regard to the profile of PFN2 expression between different histologic types gathered from the Chinese Han and Kazakh groups (Table 3).

**Table 2:** PFN2 protein expression during cancer progression by IHC analysis in Han population

Cancer progression	Immunostaining		P value
	Low (%)	High (%)	
Normal esophageal epithelium^①^	124 (91.9)	11 (8.1)	①:② P < 0.001*; ①:③ P < 0.001*
Low grade intraepithelial neoplasia^②^	31 (59.6)	21 (40.4)	②:③ P < 0.001*; ②:④ P < 0.001*
High grade intraepithelial neoplasia^③^	3 (9.0)	30 (91.0)	③:④ P = 0.241
ESCC^④^	33 (17.2)	159 (82.8)	①:④ P < 0.001*

* P < 0.05, as determined by Pearson’s χ^2^ test

**Table 3:** Relationship of PFN2 expression between Kazakh and Han ESCC tissues

Marker	Han ethnic (n = 192)		Kazakh ethnic (n = 168)		P value*
	No.	%	No.	%	
PFN2					0.282
Nondysregulated	33	17.2	22	13.1	
Dysregulated	159	82.8	146	86.9	

* Pearson’s χ^2^ test for comparison between groups

Furthermore, we examined the expression of PFN2 in 11 matched pairs of ESCC and adjacent normal tissue by Western blot. As shown in Fig. 2d, e, in all cases, PFN2 expression in the ESCC tissue was visibly higher than in adjacent normal tissue from the same ESCC patient (P < 0.01), which was consistent with the findings in IHC with respect to the PFN2 expression.

### PFN2 expression is associated with lymph node metastasis and invasion depth in ESCC

To ascertain whether the protein expression levels of PFN2 were indicative of the development state of ESCC, we further investigated the relationship between PFN2 expression and clinicopathological characteristics in two independent groups of patients with ESCC. As summarized in Table 4, in Han patients, a high PFN2 expression more frequently occurred in ESCC tissue with deep invasion (P = 0.004), and a positive lymph node metastasis occurred in Kazakh patients (P = 0.010). However, no significant correlation between PFN2 expression and other clinicopathological variables, such as gender, age, differentiation, and TNM stage, were found among the Chinese Han and Kazakh groups.

**Table 4:** Correlation between PFN2 expression and clinicopathologic features in ESCC from Han and Kazakh population

Variables	PFN2 expression in Han ethnic				PFN2 expression in Kazakh ethnic			
	Total cases	Low n (%)	High n (%)	P value	Total cases	Low n (%)	High n (%)	P value
Gender				0.817				0.173
Male	137	23 (16.8)	114 (83.2)		108	17 (15.7)	91 (84.3)	
Female	55	10 (18.2)	45 (81.8)		60	5 (8.3)	55 (91.7)	
Age (years)				0.658				0.916
<59	98	18 (18.4)	80 (81.6)		101	13 (12.9)	88 (87.1)	
≥59	94	15 (16.0)	79 (84.0)		67	9 (13.4)	58 (86.6)	
Differentiation				0.162				0.305
Well	67	15 (22.4)	52 (77.6)		39	7 (17.9)	32 (82.1)	
Moderate + poor	125	18 (14.4)	107 (85.6)		129	15 (11.6)	114 (88.4)	
Invasion depth				0.004*				0.393
T1–T2	79	21 (26.6)	58 (73.4)		83	9 (10.8)	74 (89.2)	
T3–T4	113	12 (10.6)	101 (89.4)		85	13 (15.3)	72 (84.7)	
Lymph node metastasis				0.945				0.010*
No	127	22 (17.3)	105 (82.7)		79	16 (20.3)	63 (79.7)	
Yes	65	11 (16.9)	54 (83.1)		89	6 (6.7)	83 (93.3)	
TNM stage				0.822				0.796
I + II	154	26 (16.9)	128 (83.1)		111	14 (12.6)	97 (87.4)	
III + IV	38	7 (18.4)	31 (81.6)		57	8 (14.0)	49 (86.0)	

* P < 0.05, as determined by Pearson’s χ^2^ test

### Upregulated PFN2 expression predicts unfavorable prognosis in ESCC patients

**Fig. 3:**
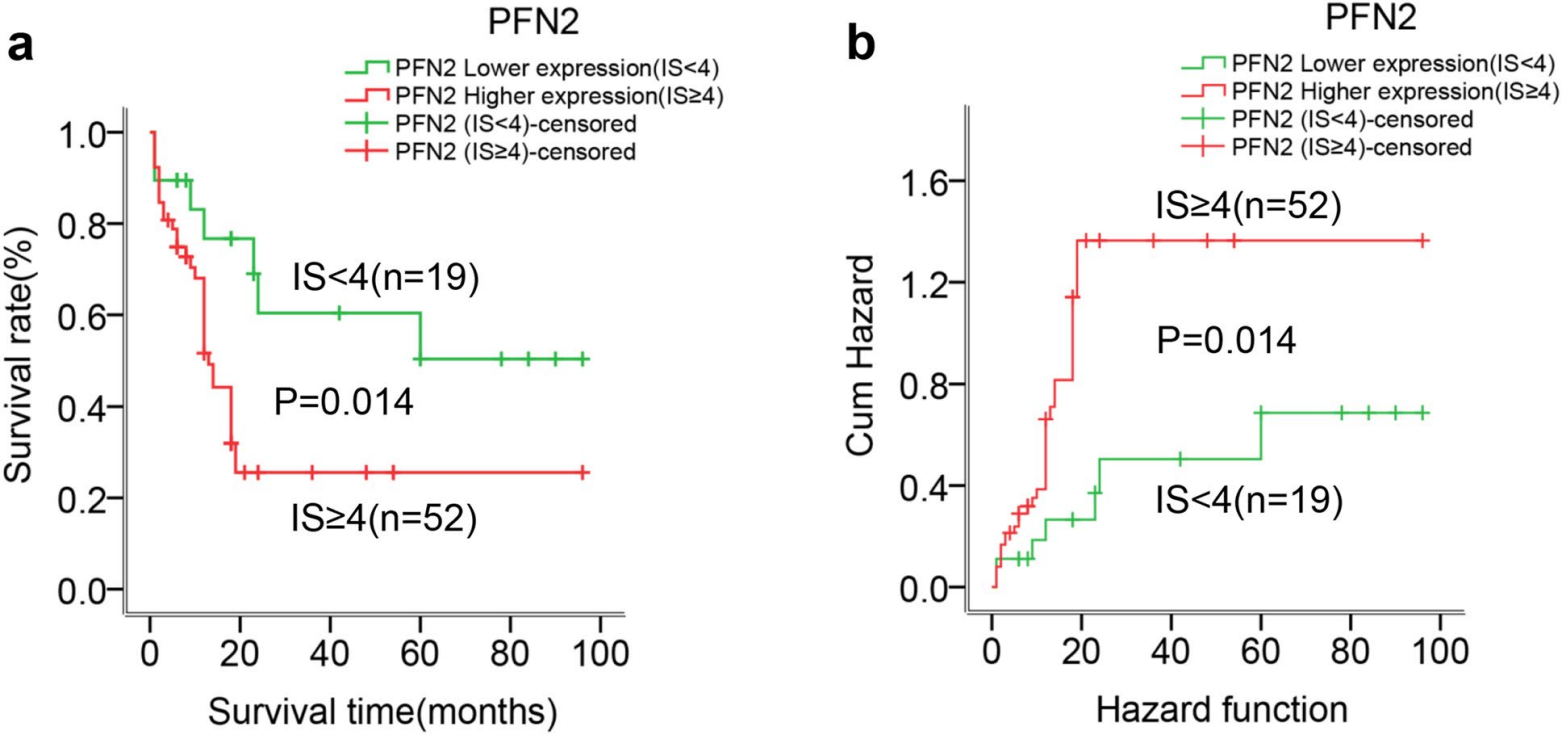
Kaplan–Meier survival curves for patients with high PFN2 expression and those with low expression. **a** ESCC patients with high PFN2 expression (IS ≥ 4) experienced a significantly shorter survival period after surgery than those with low PFN2 expression (IS < 4) (P < 0.05). **b** Patients with high PFN2 expression had a greater risk of death than those with low PFN2 expression (P < 0.05)

To assess the value of PFN2 for the prognosis of postsurgical ESCC patients, the relationship between PFN2 expression and OS of ESCC was analyzed using Kaplan–Meier method. The median survival time for patients with high PFN2 expression was 12 months (range, 1–96 months) and 42 months (range, 1–96 months) for patients with low PFN2 expression, which indicated that ESCC patients with high PFN2 expression had significantly shorter OS rates and greater risk of death than those with low PFN2 expression (log-rank test, χ2 = 5.203, P = 0.023) (Fig. 3a, b).

In addition, Cox proportional hazards modes were utilized to identify independent prognostic factors for ESCC survival. A univariate Cox analysis revealed that PFN2 protein level (HR = 2.669, 95 % CI = 1.147–6.209, P = 0.023), together with invasion depth (HR = 3.085, 95 % CI = 1.079–8.819, P = 0.036) and TNM stage (HR = 2.981, 95 % CI = 1.380–6.437, P = 0.005), was responsible for OS of ESCC patients (Table 5). Furthermore, in a multivariate analysis of significant factors, only PFN2 protein level (HR = 2.465, 95 % CI = 1.020–5.955, P = 0.045) and TNM stage (HR = 3.037, 95 % CI = 1.094–8.833, P = 0.033) were independently associated with OS of ESCC (Table 5). Overall, the data indicated that the PFN2 overexpression independently predicted a worse prognosis for ESCC patients.

**Table 5:** Univariate and multivariate Cox regression analyses of the prognostic variables in ESCC patients

Variables	Univariate analysis				Multivariate analysis			
	HR	95 % CI		P value	HR	95 % CI		P value
PFN2 expression (IS ≥ 4)	2.669	1.147	6.209	0.023*	2.465	1.020	5.955	0.045*
Gender (female)	1.734	0.910	3.303	0.094	1.574	0.780	3.176	0.206
Age (≥59 years)	0.882	0.483	1.870	0.950	1.027	0.481	2.191	0.945
Differentiation (well)	0.739	0.339	0.394	1.385	0.610	0.316	1.177	0.140
Invasion depth (T2–T3)	3.085	1.079	8.819	0.036*	1.087	0.271	4.368	0.906
Lymph node metastasis (yes)	1.147	0.590	2.228	0.686	1.198	0.595	2.415	0.613
TNM Stage (III + IV)	2.981	1.380	6.437	0.005*	3.037	1.094	8.833	0.033*

*HR* hazard ratio, *CI* confidence interval, * P < 0.05

### PFN2 may be a feasible diagnostic bioindicator for ESCC and ESIN

**Fig. 4:**
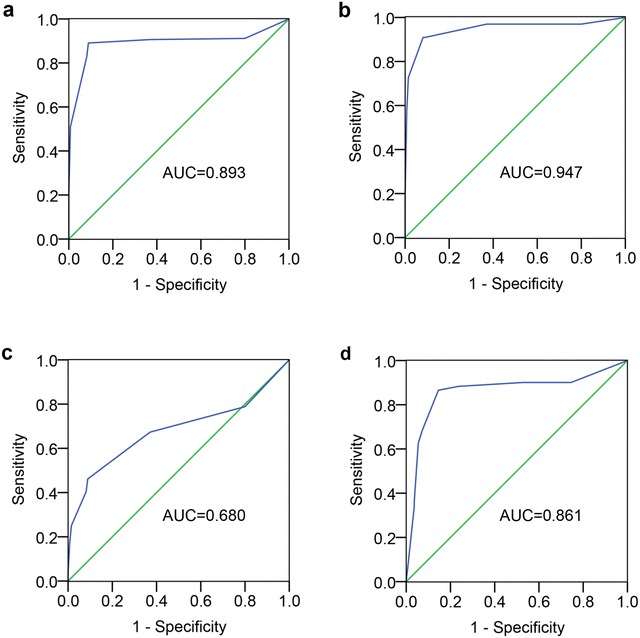
ROC curve analysis of the PFN2 IHC scores for detecting precancerous lesions and ESCC tissues. **a** ESCC tissues, **b** HGIN tissues, and **c** LGIN tissues of the Chinese Han population and **d** ESCC tissues of the Kazakh population from the controls. The area under the curve (AUC) is 0.893, 0.947, 0.743, and 0.860, respectively

Using the adjacent normal mucosa as control, the ROC curves for distinct types of tissue elucidated the point on the curve closest to (0.0, 1.0), which maximizes both sensitivity and specificity for ESCC, HGIN, and LGIN. Using the IHC cut-off scores of PFN2 as a proposed standard, the ESCC, HGIN, and LGIN tissue were distinguished easily from the control. In the Chinese Han populations, in terms of cut-off score of 4, the sensitivity and specificity values for ESCC were 96.6 and 63.0 %. HGIN and LGIN were analyzed to obtain sensitivity and specificity values of 97.0 and 63.0 %, as well as 67.3 and 63.0 %, respectively. In the Kazakh group, the sensitivity and specificity values for ESCC were 90.1 and 57.3 %, respectively (cut-off score of 4) (Fig. 4, Additional file 1: Table S1). These findings support the conjecture that PFN2 may be a feasible diagnostic bioindicator for ESCC and ESIN.

### PFN2 induces EMT phenotype and promotes the migration and invasion of ESCC cell lines

**Fig. 5:**
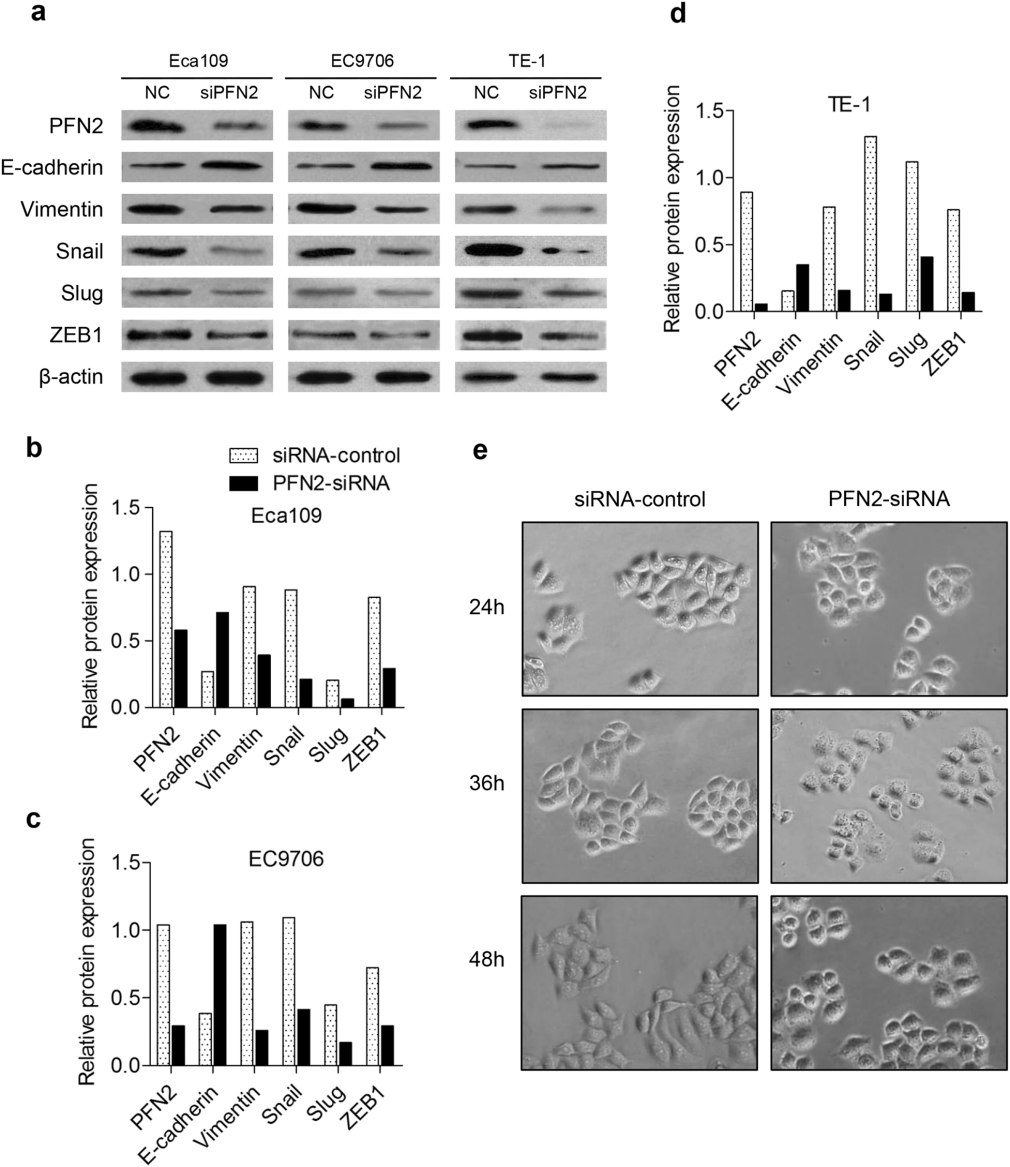
Change in EMT phenotype and morphology after PFN2-siRNA transfection in ESCC cells. **a** After silencing of PFN2 with PFN2-siRNA in ESCC cells, the protein levels of E-cadherin, Vimentin, Snail, Slug and ZEB1 were measured by Western blot. (*Bar chart* showing) Relative protein levels of E-cadherin, Vimentin, Snail, Slug and ZEB1 normalized to β-actin in **b** ECa109 cells, **c** EC9706 cells and **d** TE-1 cells. **e** Morphological change images of ECa109 cells after transfecting with PFN2-siRNA for 24, 36, and 48 h

EMT contributes to tumor invasion and metastasis in various cancers; therefore, we evaluated whether essential EMT-related markers were altered in our model. From the results of Western blot, we observed an efficient knockdown of PFN2, with over 50 % decrease in protein level in the PFN2-siRNA transfected ESCC cells, and the upregulated epithelial marker E-cadherin and significantly decreased mesenchymal markers: Vimentin, Snail, Slug and ZEB1 in Eca109, EC9706, and TE-1 cells with knockdown of PFN2 compared with the control groups (Fig. 5a, b, c, d). The Western blot results suggested that PFN2 could regulate the molecular changes of EMT in ESCC cells.

**Fig. 6:**
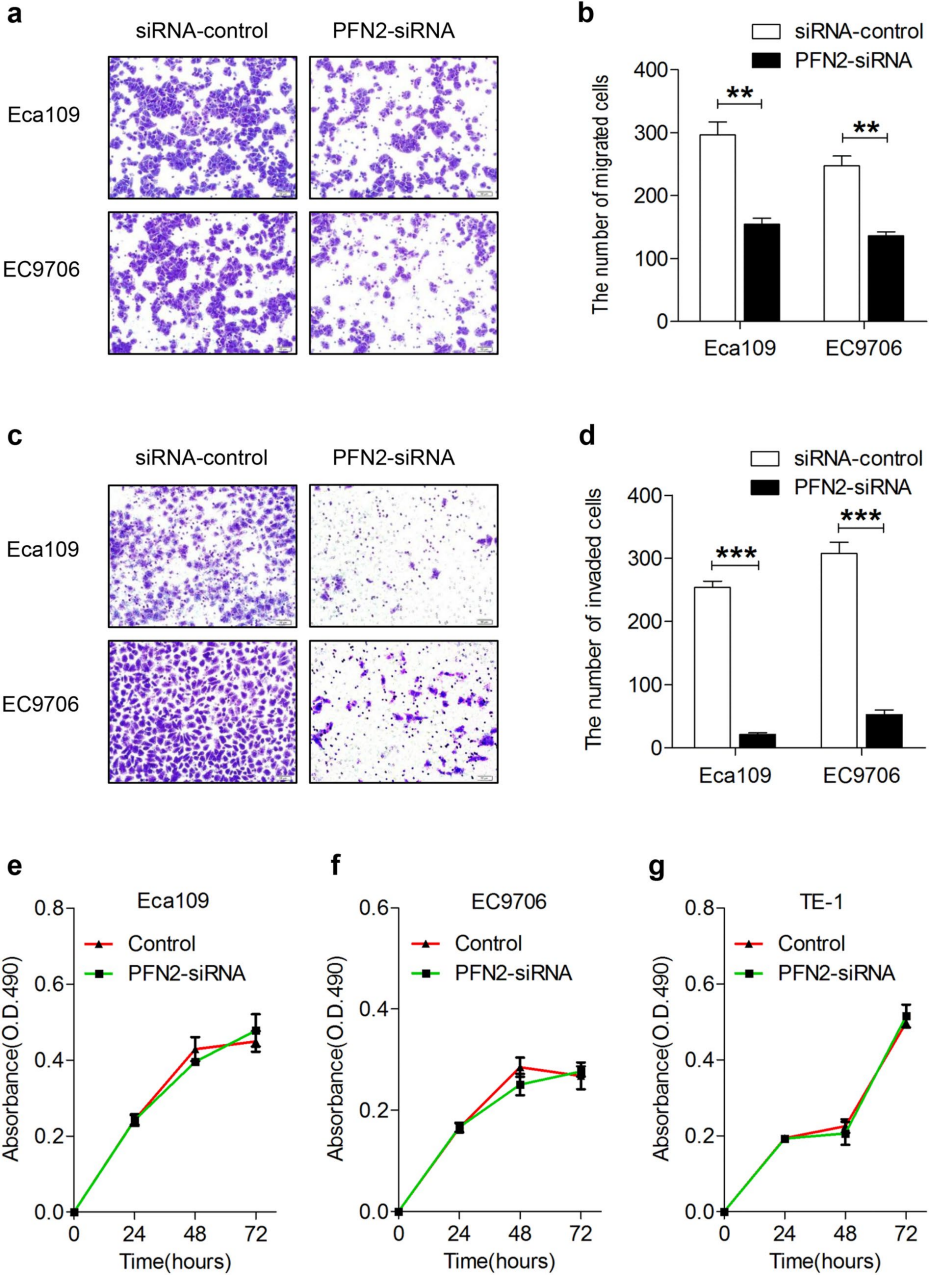
Alteration of migratory, invasive, and proliferative abilities for ESCC cells after PFN2-siRNA transfection. **a** Representative images and **c** display of migration and invasion of ECa109 and EC9706 cells transfected with PFN2-siRNA or siRNA-Control and the quantitative analysis are shown in **b** and **d**, respectively. **b** (*Bar chart*) and **d** (data) Mean ± SD of three biological replicates (**P < 0.01, ***P < 0.001). The quantitative analysis of proliferative capability of ECa109, EC9706, and TE-1 cells transfected with PFN2-siRNA or siRNA-Control is exhibited in **e**, **f**, **g**. Plots are represented as mean ± SD of data from three independent experiments

Our data on clinical samples showed that PFN2 expression is positively associated with invasion depth and lymph node metastasis of ESCC patients. Hence, we wanted to determine whether PFN2 is essential for ESCC cell migration and invasion in vitro. After silencing of PFN2, Eca109 cells exhibited a morphological change from an elongated fibroblastic phenotype to an epithelial cobblestone phenotype, which indicated that EMT weakens (Fig. 5e). Subsequently, Transwell assays showed that depletion of PFN2 significantly suppressed the migration and invasion of Eca109 and EC9706 cell lines. As shown in Fig. 6a, b, the number of cells that traversed the membrane was significantly decreased in PFN2-siRNA-treated groups compared with that in the control groups (P < 0.01). Similarly, the number of cells that invaded the Matrigel membrane was also significantly decreased in PFN2-siRNA-treated groups compared with that in the control groups (P < 0.01) (Fig. 6c, d). These results were consistent with the statistical results of clinical specimens, indicating that PFN2 is essential for the migrant and invasive properties of ESCC cells. Taken together, PFN2 may promote the migration and invasion of ESCC cell lines by inducing EMT phenotype.

## Discussion

Accumulating evidence suggests that PFN2 may be linked with multiple cancers. However, the roles of PFN2 in the reported tumors were inconsistent. In OSCC, PFN2 serves as a negative regulator of tumor growth and aggressiveness [33], whereas PFN2 significantly increases migration and invasion ability of lung cancer and colorectal cancer stem cells [19, 34]. However, whether PFN2 serves a similar role in the progression of ESCC is currently unknown. For the first time, we found that the PFN2 expression in the ESCC tissues from Chinese and Kazakh ESCC patients both increased similar to that in their ESIN tissues. Moreover, in vitro assays revealed the biological role of PFN2 in facilitating the migration and invasion of cancer cells by regulating the EMT phenotype of ESCC, thereby proving that PFN2 might be a potential biomarker for the diagnosis and a therapeutic target for the treatment of ESCC.

As a highly aggressive tumor, ESCC involves a multi-stage process, in which NEE follows a series of histological and genetic progression, goes through noninvasive precursor lesions, and finally becomes an invasive cancer [38]. Many reports indicate that precursor lesions have prognostic significance for ESCC because dysplastic lesions are frequently encountered in ESCC tissues [39]. Therefore, the expression of proteins in precursor lesions is usually predictive of ESCC progression. We found that the overexpression of PFN2 was positively related with the progression of ESCC. In Han patients, we observed that the PFN2 protein expression progressively increased from NEE to LGIN, to ESCC, peaking in HGIN, which indicated that PFN2 would soon increase once the esophageal squamous epithelium transformed into ESCC malignant progression. Patients with mild, moderate and severe dysplasia as well as in situ carcinoma have an increased risk of developing aggressive ESCC [40]. Similarly, the PFN2 protein expression was significantly higher in Kazakh ESCC than in NEE. Although the two groups had different ethnic customs and dietary histories, high level of PFN2 protein was equally observed in both Han and Kazakh ESCC tissues. Therefore, we confirmed the notion that PFN2 might function as an oncogene of ESCC. As far as we concerned, this is the first report that exhibits high level of PFN2 protein inamongst ESCC patients from Chinese Han and Kazakh populations. A similar phenomenon was observed in ESIN tissues.

To investigate the feasibility of using PFN2 as a clinical diagnostic biomarker of ESCC and ESIN, we constructed ROC curves to assess the specificity and sensitivity of PFN2 in different types of tissues. We acquired high sensitivity and specificity values that underscored the potential availability of PFN2 as a novel diagnostic biomarker. Notably, our initial data were only derived from immunohistochemical results, which constituted a sensitive and cost-efficient method for estimating protein expression [41]. This method has also been used to recognize several potential diagnostic markers and therapeutic targets [42]. Therefore, other protein analytical methods should be used to further confirm the immunohistochemistry-based results that are presented in this paper. In addition, we found that PFN2 expression influenced the survival of ESCC patients, with overexpression of PFN2 being associated with worse OS. Cox regression analysis revealed that the PFN2 expression could serve as an independent prognostic factor for ESCC. The relations between PFN2 expression and worse OS have been previously analyzed in several tumors. Previous studies also proposed that abnormal PFN2 expression could predict poor prognosis [19, 33]. Our results support the theory that abnormal PFN2 expression may serve as a prognostic biomarker, which in turn highlights the implication that an increased PFN2 expression can be an early indicator of high-risk population and prognostic biomarker.

Another important finding in this study was found that PFN2 expression was positively associated with invasion depth amongst Han patients with ESCC, as well as lymph node metastasis amongst Kazakh ESCC patients. Therefore, PFN2 overexpression may be important for the development of ESCC and can promote the metastasis and aggressiveness of ESCC. Nevertheless, the inconsistent conclusions from these two groups might be attributed to the restrictions in the sample size, the heterogeneity of the population and their different hereditary backgrounds. Therefore, these findings should be explored further in future studies by using uniform ethnic cohorts and employing a larger sample size. Our findings are compatible with those of several reports on PFN2 in other types of tumors. For instance, one research showed that PFN2 mRNA levels and protein were significantly up-regulated in lung cancer tissues, whilst PFN2 overexpression strongly promoted lung cancer migration and invasion [19]. Another study reported that up-regulated PFN2 expression in colorectal cancer stem cells enhanced the ability of migration and invasion [34, 43]. By contrast, Ma et al. observed a decreased PFN2 protein expression in OSCC tissues and suggested that the down-expression of PFN2 in OSCC tissues was significantly associated with vascular invasion and advanced TNM stage [33]. These contradictory results might be attributed to the heterogeneity of the tumor and the limited sample size. Moreover, PFN2 molecules could bind tightly with a proline-rich motif, and various proteins contain one or more proline-rich motifs, such as Ena/VASP proteins (Mena, VASP and EVL) [44–46], Arp2/3 complex [47] and WASP [48]. Therefore, PFN2 may be involved in the regulation of different signaling pathways, such as PI3K–AKT [49] and TGF-β/Smad signaling [19], thereby leading to different biological consequences. Therefore, further research must elucidate the association of PFN2 expression with the invasion and metastasis of ESCC by employing a larger sample.

In epithelial cancers, EMT is regarded as one of the major molecular mechanisms that promote invasion and metastasis [50, 51]. Loss of E-cadherin is a fundamental step of EMT by which polarized epithelial cells lose the tight cell–cell junction and enhance the migratory capacity. In our study, E-cadherin was clearly up-regulated by the knockdown of PFN2. This result was in agreement with the decreased migration and invasion abilities of ESCC cells. Some reports showed that several EMT-inducing transcription factors, such as Snail, Slug and ZEB1, could directly or indirectly repress E-cadherin to promote invasion and metastasis [52, 53]. Snail and Slug could bind to the E-box hexa-nucleotide DNA motif in the human E-cadherin promoter by a resembling structure with a highly conserved carboxy-terminal domain [54]. ZEB1 induces EMT by down-regulating E-cadherin and up-regulating a range of mesenchymal markers, such as N-cadherin, Vimentin and MMPs, thereby promoting cell migration, invasion and metastasis [55]. In our study, silencing PFN2 significantly suppressed the expressions of Snail, Slug and ZEB1. These results were consistent with those of previous lung cancer studies [19]. In lung cancer, PFN2 has been identified as a critical activator of TGF-β/Smad signal-inducing EMT. Similarly, another mesenchymal marker, Vimentin, was obviously downregulated by the knockdown of PFN2. Vimentin is a filament that is highly expressed in mesenchymal cells and is generally used to determine those tumor cells that are undergoing EMT based on the positive association of Vimentin expression with the invasion and metastasis in multiple cancers [56]. Notably, our results were accorded with previous ESCC studies [57]. Pang et al. were the first to report that the TGF-β1/Smad signaling pathway could induce EMT in ESCC. Unsurprisingly, the silencing of PFN2 significantly reversed the fibroblastoid-like phenotype of ESCC cells. In other words, the depletion of PFN2 suppressed the invasion and metastasis of ESCC cell lines as well as induced the reversion of EMT, which suggested that PFN2 enhanced the invasion and metastasis of ESCC cell lines by inducing EMT. We contend that PFN2 may promote invasion and metastasis by activating the TGF-β1/Smad signaling pathway inducing EMT in ESCC, but this contention must be clarified further.

However, it must be pointed out that, in the present study, we adopted an approach similar to that of Valenzuela–Iglesias et al. [58]. Specifically, we detected the PFN2 expression as a whole and did not further distinguish the isoforms of PFN2. Therefore, the differences between PFN2a and PFN2b in terms of ESCC progression should be clarified in future studies.

## Conclusions

In summary, our clinical study has characterized PFN2 as a molecular marker for ESCC progression and metastasis, and more importantly, our study has identified an independent prognostic factor for ESCC patients, indicating that PFN2 could serve as an early detector of ESCC high-risk population and a novel prognostic marker for ESCC. Moreover, we found knockdown of PFN2 significantly reduced migration and invasion by mediating EMT phenotype in vitro. Targeting PFN2 may offer a promising therapeutic strategy for ESCC treatment.

## Additional file

10.1186/s12967-016-0884-y The high sensitivity, specificity and AUC values of PFN2 in ESCC, HGIN, and LGIN.

## Abbreviations

ESCC: esophageal squamous cell carcinoma
PFN2: profilin 2
IHC: immunohistochemistry
EMT: epithelial–mesenchymal transition
NEE: normal esophageal epithelium
LGIN: low-grade intraepithelial neoplasia
HGIN: high-grade intraepithelial neoplasia
ESIN: esophageal squamous intraepithelial neoplasia
OS: overall survival
OSCC: oral squamous cell carcinoma
TNM: tumor node metastasis
TMA: tissue microarray
HE: hematoxylin and eosin
IS: immunohistochemistry score
ROC: receiver operating characteristic
