# Supplementary material for: PFN2, a novel marker of unfavorable prognosis, is a potential therapeutic target involved in esophageal squamous cell carcinoma
Source: J Transl Med. 2016 May 17;14:137. doi: 10.1186/s12967-016-0884-y (PMC4870769; doi:10.1186/s12967-016-0884-y)
Supplement: Supplementary file 1 — 10.1186/s12967-016-0884-y The high sensitivity, specificity and AUC values of PFN2 in ESCC, HGIN, and LGIN. [file 12967_2016_884_MOESM1_ESM.docx]

**Additional file 1: Table S1. The high sensitivity, specificity and AUC values of PFN2 in ESCC, HGIN, and L****GIN**

| **Comparison** | **AUC** | **Sensitivity (%)** | **Specifity (%)** | **Cut-off value*** |
| --- | --- | --- | --- | --- |
| **ESCC versus NEE** |  |  |  |  |
| **Han ethnic** | 0.893 | 90.60 | 63.00 | 4 |
| **Kazakh ethnic** | 0.861 | 90.10 | 57.30 | 4 |
| **HGIN versus NEE** | 0.947 | 97.00 | 63.00 | 4 |
| **LGIN versus NEE** | 0.680 | 67.30 | 63.00 | 4 |

**^a^** Cut off level was set to provide optimal sensitivity and specificity.
